# Symptom burden and health-related quality of life impacts of smoldering multiple myeloma: the patient perspective

**DOI:** 10.1186/s41687-020-00253-2

**Published:** 2020-11-09

**Authors:** Milenka Jean-Baptiste, Katharine S. Gries, William R. Lenderking, John Fastenau

**Affiliations:** 1 Evidera, 7101 Wisconsin Avenue #1400, Bethesda, MD 20814 USA; 2 Janssen Pharmaceutical Patient Reported Outcomes, 1000 US-202, Raritan, NJ 08869 USA; 3 Evidera, 500 Totten Pond Road, Waltham, MA 02451 USA

**Keywords:** Smoldering multiple myeloma, Oncology, Patient-reported outcomes, Health-related quality of life

## Abstract

**Background:**

Smoldering multiple myeloma (SMM) is an early form of multiple myeloma (MM). SMM is typically considered asymptomatic, and research on how it affects health-related quality of life (HRQoL) is limited. This study assessed the symptoms and HRQoL of patients with SMM and those who progressed from SMM to MM and evaluated the content validity of two patient-reported outcome instruments (EORTC QLQ-C30 and nine items from the EORTC QLQ-MY20) for use in SMM clinical trials. To address these objectives, concept elicitation and cognitive interviews were conducted with SMM patients and recently diagnosed MM patients.

**Results:**

Fifteen adult SMM and six adult MM participants with a prior SMM diagnosis were interviewed. On average, SMM study participants were 61 years old (46.0–78.0), 11 (73%) were female, and diagnosed 2.6 (±2.0) years ago. Each participant had experienced at least one symptom, most commonly tiredness/fatigue, weakness, and pain. The most common HRQoL impacts were emotional and physical. SMM study participants demonstrated good understanding of both the EORTC QLQ-C30 and EORTC QLQ-MY20 subscales and found them relevant to their SMM health state. The average age of MM participants was 53 years old (39.0–62.0); 5 (83%) were female and diagnosed 1.9 years ago (±2.1). MM participants most commonly reported tiredness, weakness, constipation, shortness of breath, and dry mouth as occurring when they progressed from SMM to MM.

**Conclusions:**

Although previously described as asymptomatic, these SMM participants reported experiencing symptoms that affected their lives. Additionally, the EORTC subscales measured symptoms SMM patients experienced. The participants with MM reported that the symptom burden and HRQoL impacts increased when diagnosed with MM. These findings suggest the need for increased surveillance of symptoms within the SMM population and further suggest that the EORTC subscales can be used to assess symptoms and impacts in both the SMM and MM populations.

## Background

Smoldering multiple myeloma (SMM) is a clonal plasma cell disorder [10]. This rare blood cancer is the precursor to multiple myeloma (MM) [8]. SMM incidence is estimated at 0.9 cases per 100,000 people; the median age of diagnosis is 67 years [2, 11]. Considered the intermediate stage between monoclonal gammopathy of undetermined significance (MGUS) and MM, SMM is more likely to progress to MM as compared to MGUS [6]. The risk of MM progression for SMM patients has been classified into three categories: low risk of progression within 5 years, intermediate risk reflecting a 40% probability of progression within 5 years, and high risk, with a 50% probability in 2 years [7]. Currently, a patient’s risk level dictates the treatment they receive. SMM patients who have low risk of progressing to MM should not receive treatment, while those at high risk should [2]. Patient-reported outcome (PRO) instruments assessing health-related quality of life (HRQoL) may be useful in understanding the burden of disease and the patients’ health state for all SMM risk levels, particularly for those at lower risk levels.

Although new research on SMM treatments is emerging, little is published about the experiences of patients diagnosed with SMM and their HRQoL. PRO instruments to measure how a patient functions and feels can be used in clinical trials or in clinical practice alongside clinical efficacy measures (e.g., progression-free or overall survival) to help clinicians and patients make informed and comprehensive decisions regarding the best available treatments [3]. Validating a PRO instrument in the SMM population is needed before they can be used to inform providers about the presence of symptoms and impacts on the patient.

A number of studies have assessed HRQoL and the use of PRO instruments as they relate to MM [5, 12]. For example, the European Organisation for Research and Treatment of Cancer core-30 item scale (EORTC QLQ-C30) and the multiple myeloma 20-item module (EORTC QLQ-MY20 )[4, 15] are reliable and valid instruments for assessing HRQoL, symptoms, and functioning in the MM population [4]. However, as SMM is often considered asymptomatic, limited research has been conducted to assess SMM patients’ HRQoL and the validity of PROs in the this population.

This study aimed to conduct interviews with patients diagnosed with SMM, prior to disease progression and treatment for MM, to identify any symptoms and impacts important to them. This study also assessed the overall experience and symptoms of those transitioning from SMM to MM. A secondary study objective was to assess the content validity of the EORTC QLQ-C30 and the two subscales in the EORTC QLQ-MY20 in patients with SMM.

## Methods

This cross-sectional, qualitative study involved telephone-based, single session interviews with participants diagnosed with SMM and MM. Qualitative interviews were selected as the mechanism for data collection as they allowed a forum for participants to individually express and share their experiences with SMM and MM. They also allowed for a space for the team to individually assess participant comprehension of a PRO instrument without being influenced by other participant experiences.

Participants were mailed a study packet containing the informed consent, the PRO instruments, and sociodemographic form. Prior to the start of the interview, the informed consent form was reviewed, participants were given a chance to ask questions, and consent was obtained. At any point throughout the interview, participants had the opportunity to withdraw their participation without being penalized. The semi-structured interviews then asked about their symptoms related to their health state and the effects on daily life. SMM participants then completed the paper version of the self-administered PRO instruments before being probed on their understanding of questionnaire items and the appropriateness of each question. At the completion of the interview participants were instructed on how to mail back the completed PRO instruments. Those in the MM cohort were asked specific questions about what symptoms they experienced as they progressed from SMM to MM.

No investigational drugs, devices, or invasive procedures were administered or evaluated as part of this study. A protocol and semi-structured interview guide were developed and approved by Quorum, an institutional review board in October 2017.

### Recruitment and eligibility criteria

In order to reach a saturation of concepts during the concept elicitation interviews the target sample size was 21 participants, with the majority being participants diagnosed with SMM. Participants were recruited from five clinical research sites from the Northeastern, Northwestern, and Southeastern regions of the United States. The clinical sites included a research facility (*n* = 1), private practices (*n* = 2), and hospitals (n = 2). As SMM is rare, additional participants were recruited with the help of a recruitment vendor, The Nielsen Company LLC. Both the clinical sites and the recruitment vendor were similarly trained in study procedures and recruited both SMM and MM using the same procedures. Participants were identified using two methods. The first method involved searching through their databases to identify participants who met the eligibility criteria. Based on this initial screening, participants were then called using an IRB-approved recruitment and screening script to discuss the study objective and procedures. The second method involved posting an IRB-approved flyer to listservs or web-based patient boards. Participants would then call or email the study specific phone line/ email inbox to find out more about the study. Once the participant indicated interest in the study, an interview was scheduled.

To be eligible, SMM participants needed to have a clinician-confirmed diagnosis with measurable signs of SMM but not receiving treatment for MM. SMM participants also had to be 18 years or older, diagnosed with SMM at least 30 days before screening, and able to participate in an interview lasting up to 90 min. Participants were excluded if they had a history of malignancy other than SMM 1 year prior to the date of screening, or if they had evidence of end-organ damage (e.g hypercalcemia, renal failure, anemia, and bone lesions) [9] or a myeloma-defining event (MDE). Members of the MM cohort had to be at least 18 years old to participate in this study. They needed a clinician-confirmed diagnosis of MM within the last 5 years with a prior confirmed SMM diagnosis. The MM diagnosis had to be at least 30 days prior to screening for this study.

### Procedures and PRO instruments

Interviews were conducted as: (1) open-ended concept elicitation and (2) cognitive interviewing. The concept elicitation interviews were firstly informative of whether patients with a SMM diagnosis have symptoms and then secondarily which symptoms they experienced. The interviews began with study participants providing general background information regarding SMM, such as how they initially found out about their condition. Participants were then asked to report the symptoms they experienced since their diagnosis, including any impacts SMM had on their daily lives. If not already mentioned, the interviewer probed on known symptoms of MM and impacts commonly experienced by patients with cancer. The cognitive interviewing portion of the interviews assessed patient interpretation of the instructions, items, response options, and recall period on the EORTC QLQ-C30 and EORTC QLQ-MY20 to assess their content validity for this target population.

The EORTC QLQ-C30 (version 3) has demonstrated reliability, validity, and clinically-meaningful change among patients with MM [1, 15, 16]. The EORTC QLQ-MY20 was validated for use with the EORTC QLQ-C30 [4]. For the current study, nine of the 20 items from the EORTC QLQ-MY20 (Disease Symptoms and Future Perspective subscales) were selected for cognitive debrief. These two scales (the nine items) were selected based on a review of the clinical literature because these concepts appeared to be more relevant with newly diagnosed patients who had not received prior MM treatment and patients who would be dealing with the emotional burden of a SMM diagnosis.

Additionally, participants were asked how the concepts on these instruments related to their SMM experience and whether relevant concepts were missing. Participants also completed basic descriptive information (e.g. gender, age, education level) to describe the study population.

### Analysis

Demographic and clinical data were summarized to characterize the study sample. All interviews were audio-recorded, transcribed, and analyzed using ATLAS.ti version 7.5 or 8, a qualitative analysis software. A coding dictionary was developed to ensure consistency between the 2 trained coders who were part of the study team. In addition to being trained on the coding dictionary, coders were asked to code the same interview transcript and any discrepancies in coding were discussed. Data were analyzed using an open coding approach [14], where key themes were identified that described important concepts raised by the participants.

Participant quotes were grouped by thematic code to assess saturation of concepts. Saturation was reached when the inclusion of additional study participants did not provide any substantially new or previously unrecognized issues or concepts. Thus, responses from these individual interviews were analyzed with the goal of evaluating the amount of novel information that was observed in each interview. Verbatim transcripts were also reviewed with the goal of demonstrating when saturation was achieved through the use of a saturation table or grid that documented the number of interviews that identified a certain concept or category. After reconciling all coding, the analysis was finalized and the output, consisting of participant quotations organized by thematic code was produced.

## Results

### Study sample characteristics

A total of 15 adult SMM participants were interviewed: four participants recruited from clinical sites and 11 participants recruited by Nielsen. Full demographics are provided in Table 1. On average, study participants were 61 years old (46.0–78.0), 11 were female (73%), and diagnosed 2.6 (±2.0) years ago.

**Table 1:** Sociodemographic and clinical characteristics of study sample

Characteristic	SMM Participants(***N*** = 15)	MM Participants(***N*** = 6)
**Age (years)**		
Mean (SD)	61.4 (9.2)	53.0 (8.9)
Median (Range)	61.0 [46.0–78.0]	54.0 [39.0–62.0]
**Gender, n (%)**		
Male	4 (26.7%)	1 (16.7%)
Female	11 (73.3%)	5 (83.3%)
**Ethnicity, n (%)**		
Hispanic or Latino	1 (6.7%)	1 (16.7%)
Not Hispanic or Latino	14 (93.3%)	5 (83.3%)
**Racial Background, n (%)**		
White	5 (33.3%)	4 (66.7%)
Black or African American	7 (46.7%)	1 (16.7%)
Asian	1 (6.7%)	0 (0.0%)
Other^a^	2 (13.3%)	1 (16.7%)
**Living/Domestic Situation, n (%)**		
Living alone	1 (6.7%)	0 (0.0%)
Living with a partner or spouse, family, or friends	14 (93.3%)	6 (100.0%)
**Employment status, n (%)**		
Employed, full-time	9 (60.0%)	1 (16.7%)
Employed, part-time	0 (0.0%)	1 (16.7%)
Unemployed	0 (0.0%)	2 (33.3%)
Retired	4 (26.7%)	0 (0.0%)
Disabled	1 (6.7%)	2 (33.3%)
Other^b^	1 (6.7%)	0 (0.0%)
**Highest Level of Education, n (%)**		
Less than high school	1 (6.7%)	0 (0.0%)
Completed high school	2 (13.3%)	1 (16.7%)
Associate degree, technical or trade school	2 (13.3%)	1 (16.7%)
Some college	2 (13.3%)	1 (16.7%)
College	5 (33.3%)	2 (33.3%)
Graduate school	3 (20.0%)	1 (16.7%)
**Marital Status, n (%)**		
Married	15 (100.0%)	4 (66.7%)
Single	0 (0.0%)	1 (16.7%)
Divorced	0 (0.0%)	1 (16.7%)
**Time since SMM/MM diagnosis (years)**		
Mean (SD)	2.6 (2.0)	1.9 (2.1)
Median (Range)	2.3 [0.1–6.9]	0.7 [0.2–4.8]
**Other health conditions** ^**d**^**, n (%)**		
Allergic rhinitis	1 (6.7%)	0 (0.0%)
Anxiety	1 (6.7%)	1 (16.7%)
Arthritis	2 (13.3%)	0 (0.0%)
Cardiovascular disease	1 (6.7%)	0 (0.0%)
Hypertension (high blood pressure)	4 (26.7%)	1 (16.7%)
Kidney Disease	0 (0.0%)	1 (16.7%)
Pulmonary Disease	0 (0.0%)	1 (16.7%)
Osteoporosis	0 (0.0%)	1 (16.7%)
Other^c^	3 (20.0%)	2 (33.3%)
No other health conditions	7 (46.7%)	4 (66.7%)
**Current Treatments for SMM/ MM**^**d**^**, n (%)**		
Iron Supplement	1 (6.7%)	0 (0.0%)
Carfilzomib, Dexamethasone, Lenalidomide	0 (0.0%)	1 (16.7%)
Bortezomib, Cyclophosphamide, Decadron	0 (0.0%)	1 (16.7%)
Bortezomib, Lenalidomide, Dexamethasone, Prednisone	0 (0.0%)	1 (16.7%)
Velcade, Dexamethasone	0 (0.0%)	1 (16.7%)
No Treatment	14 (93.3%)	2 (33.3%)

^a^Other Racial background include: “Indian and White” (n = 1) and “Hispanic” (n = 2)

^b^Other Employment Status include: “Homemaker and Retired” (*n* = 1)

^c^Other Health Conditions include: “DVT, Systolic Aortic Murmur” (n = 1) and “hyperlipidemia” (n = 3), and “Chronic pain, colon cancer, neuropathy” (n = 1)

^d^Not mutually exclusive

Abbreviation: *SD* Standard deviation

A total of 7 SMM participants (46.7%) had no other health conditions, 4 participants (26.7%) had hypertension, 3 participants (20.0%) had other conditions including deep vein thrombosis, systolic aortic murmur, or hyperlipidemia, 2 participants (13.3%) had arthritis, and one participant (6.7%) each reported experiencing allergic rhinitis, anxiety, and cardiovascular disease.

A total of 6 adult MM participants were interviewed: 2 participants recruited from clinical sites and 4 participants recruited by Nielsen. On average, study participants were 53 years old (39.0–62.0), 5 were female (83%) and diagnosed 1.9 (±2.1) years ago (Table 1). Most (*n* = 4) MM participants reported no other health condition aside from MM.

### Concept elicitation on SMM patient symptoms and impacts experienced

#### SMM patient reported symptoms

**Fig. 1:**
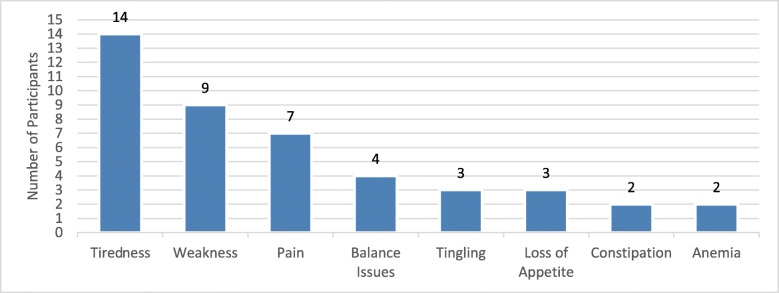
Frequency of Top SMM Symptoms Reported. Note: Headache (n = 1), weight loss (n = 1), blood clotting (n = 1), and diarrhea (n = 1) were less frequently reported as symptoms that specific participants experienced

Each participant reported at least one symptom. The commonly reported symptoms related to the SMM diagnosis were tiredness, weakness, and pain (Fig. 1). The other less commonly reported symptoms reported by one participant each were headache, weight loss, blood clot, low blood levels, and diarrhea.

Ninety-three percent of participants (*n* = 14) with SMM reported tiredness, sometimes described as feeling fatigued. Participants described tiredness as having less energy than their previous energy level or feeling that it was easier to tire. The duration of their reported tiredness ranged from half a day to a few times a week or month.

*Fatigue feels like, um, somebody’s just drained me, like somebody did reverse vacuuming, stuck a vacuum in my head and sucked out energy. It – I don’t want to get off the couch, which isn’t my normal personality (68 y.o; Female).*

Tiredness limited their ability to carry out daily activities, such as household chores, and their ability to carry out hobbies.

*I would, I just notice that I tire more easily if I do yard work, mow the yard, I come in and feel more tired than I think I did 5 years ago or so … When I go outdoors, I do snow ski, I do biking. I just probably can’t go as far as I used to go (62 y.o; Male).*

Weakness was the second most common symptom reported by 8 SMM participants (53%). Like participant descriptions for tiredness, weakness was described as having reduced energy or reduced strength. However, participants also described feeling the sensation of passing out as it related to weakness. Weakness was experienced over the entire body and would affect them ‘throughout the day’ to ‘multiple times monthly.’

*It’s like—it’s like I feel a little weak, I feel like I don’t have my strength like 100%, it might be diminished by maybe 10% or something like that (59 y.o; Male).*

Pain was the third most frequently endorsed symptom (*n* = 7, 47%). Participants reported that the pain was in their back, legs, bone, or ribs (one participant did not indicate a specific location). It should be noted that of those who described experiencing pain, 29% (*n* = 2/7) attributed it to other conditions or issues such as sciatica and a busy job. The frequency ranged from ‘half an hour’ to ‘every 2 to 3 months.’

*It aches in that it doesn’t really feel like, uh, a bruise it feels like, um, a tightness with pain I guess would be my best description (65 y.o; Female).*

#### SMM impacts on functioning and health related quality of life

**Fig. 2:**
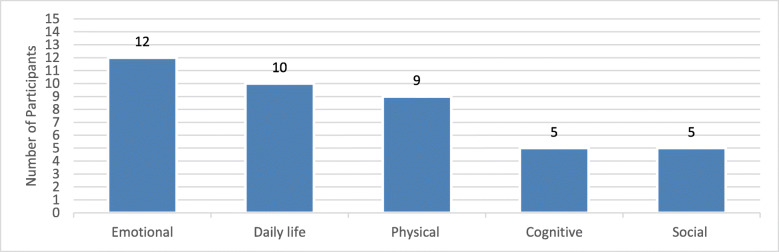
Frequency of SMM Impacts Reported. Note: One participant endorsed not having any impacts at all

A total of fourteen participants (93%) living with SMM reported HRQoL impacts. A total of 18 impacts were described and grouped into five categories: emotional, daily life, physical, cognitive, and social (Fig. 2).

**Fig. 3:**
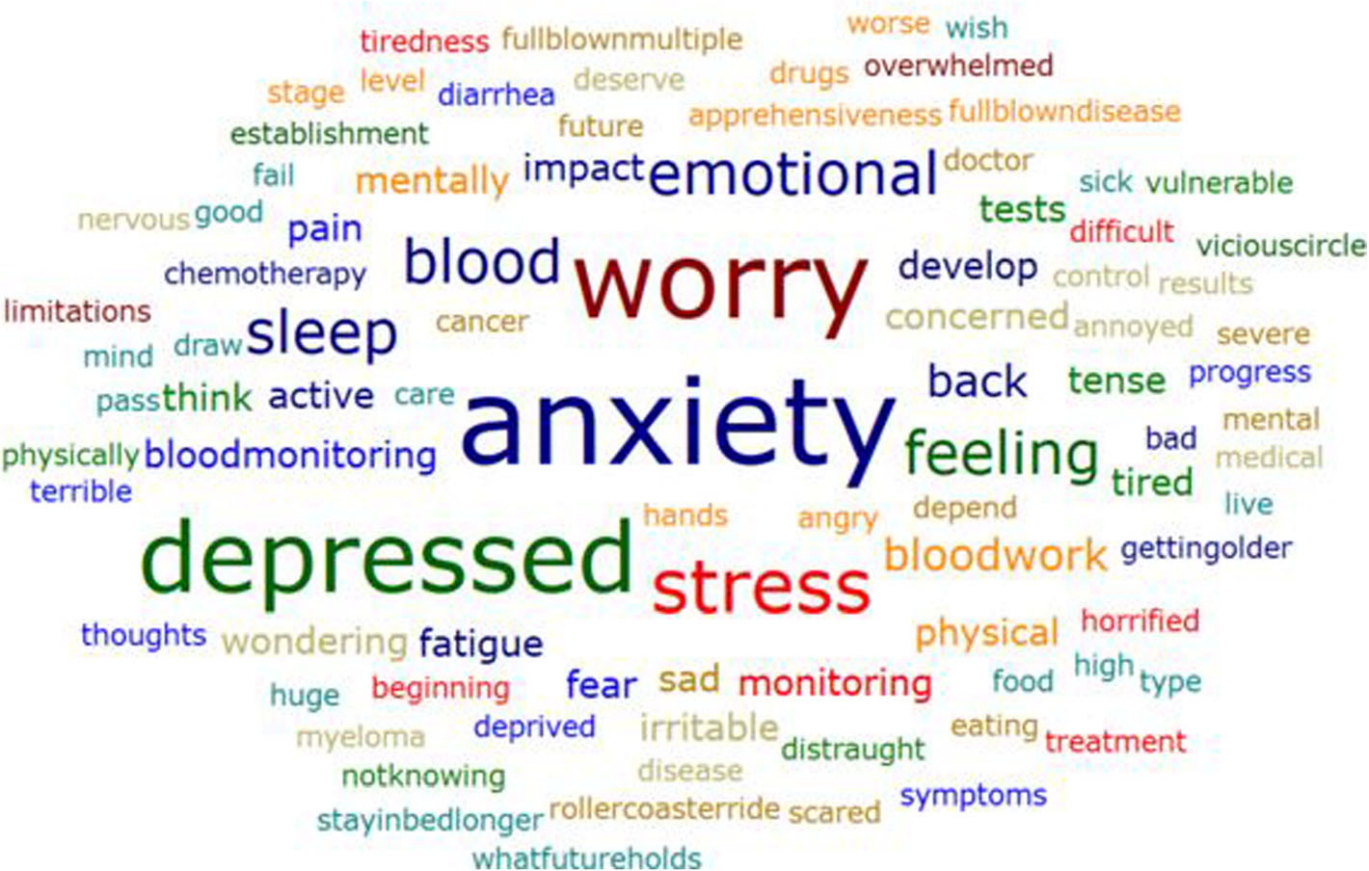
SMM Emotional Impacts Word Cloud

Nearly every participant talked about the emotional impact of being diagnosed with SMM (*n* = 12, 80%). The main concepts were worry and concern (*n* = 9/12, 75%), stress/anxiety/nervousness (*n* = 8/12, 67%), and depression (*n* = 7/12, 58%). The word cloud depicted in Fig. 3 shows the emotional concepts discussed. The size of the words indicates the frequency with which they were mentioned. As exemplified by “worry” and “anxiety,” psychological impacts were the most commonly reported impacts of this disease.

Ten participants (67%) discussed how SMM affected their daily lives. This included insomnia, the burden of frequent visits to the doctor, difficulty with memory, and limitations in self-care. Physical impacts were reported by approximately half of the participants (*n* = 9/15, 60%), limiting their ability to walk as they once did, carry heavy things, and overall reducing physical activity. Cognitive impacts, such as concentrating and memory issues, were reported to a lesser extent (*n* = 5/15, 33%). It was difficult for participants to separate getting older from the impact of SMM. Other areas of impact included social life and a change in their family life or relations with others (n = 5/15, 33%).

#### SMM transition to MM patient-reported symptoms

There were five symptoms MM participants described in both SMM and MM: tiredness/fatigue, constipation, shortness of breath, dizziness, and dry mouth. Tiredness/fatigue and constipation were the two symptoms that overlapped with symptoms reported by SMM participants.

Approximately 67% of participants (*n* = 4/6) reported that tiredness started when they had SMM and continued into MM (transitional symptom), 17% (*n* = 1/6) mentioned it started when they had SMM and stopped when diagnosed with MM, an additional 17% of participants (n = 1/6) indicated it started with MM treatment. Weakness was reported by approximately 67% the MM participants (n = 4/6) and commonly described as being part of tiredness and feeling numbness. Fifty percent of the four participants (*n* = 2/4) reporting weakness mentioned beginning to experience weakness when they were diagnosed with MM while the other 50% of participants (n = 2/4) reporting weakness did not specifically describe when it began.

*Well, it kind of feels like, you know, you’ve got a weight on your shoulders and you’re just—you’re weak. And you know, I was always—always, um, being healthy and working out and running and everything to now not exercising and not having stamina or strength (50 y.o; Female).*

While constipation, shortness of breath, and dry mouth were discussed as part of living with SMM, some participants did not attribute these symptoms to SMM/MM. These symptoms were either present prior to the SMM diagnosis and part of a chronic problem or possibly were due to comorbid conditions or medications they were taking.

### Completion of the EORTC QLQ-C30 and EORTC QLQ-MY20

Both SMM and MM participants completed the EORTC QLQ-C30 and EORTC QLQ-MY20 measures during the interview. The EORTC QLQ-C30 includes functional scales (physical functioning, role functioning, emotional functioning, cognitive functioning, and social functioning), symptom scales (fatigue, nausea and vomiting, and pain), and a Global Health Status scale. Participants responded with *‘not at all’* or *‘a little.’* Items for which participants selected *‘Quite a bit’* and *‘Very Much’* in higher frequency focused on whether their physical or medical condition impacted their health, whether participants felt they needed to rest, and whether participants felt depressed. EORTC QLQ-C30 Functional Scale scores ranged on a scale from 0 (worst health) to 100 (best health) [1].

**Fig. 4:**
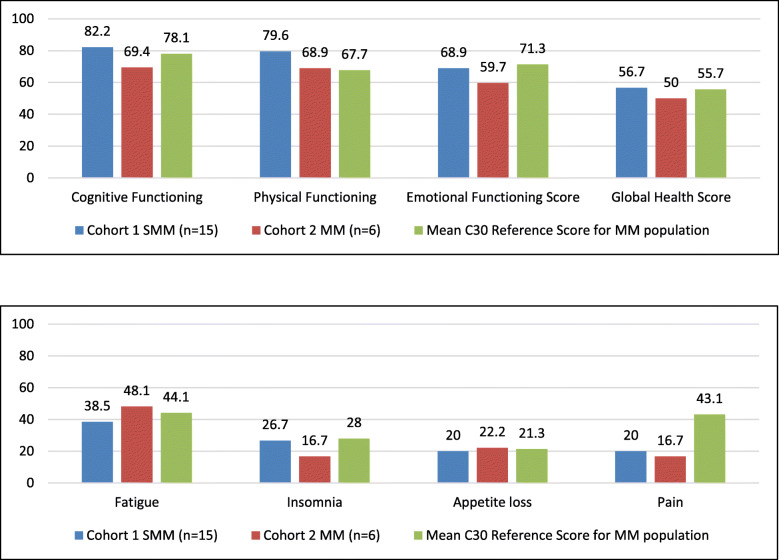
Mean EORTC QLQ C30 Summary Scores^1–2.^ 1 For Cognitive Functioning, Physical Functioning, Emotional Functioning, and Global Health Scores, higher scores reflect better health. 2 For Symptoms such as fatigue, insomnia, appetite loss, and pain higher scores reflect experiencing the symptom more often

SMM and MM participants generally scored highest on the cognitive and physical functioning sub-scales of the EORTC QLQ-C30. For cognitive functioning, participants scored above the mean EORTC QLQ-C30 MM “All Myeloma Group” reference group scores [13] by four points while for physical functioning participants scored above the mean reference group scores by 12 points. This indicates higher levels of both cognitive and physical functioning relative to the other functional scales. Overall, when compared to other functioning subscales, participants scored lower on the Global Health Status and Emotional Functioning subscales. Throughout all sub-scales SMM participants tended to consistently score marginally higher than MM participants, indicating slightly better functioning across these sub-scales (Fig. 4).

Additionally, with the exception of fatigue, the EORTC-QLQ-C30 scores for the SMM and MM populations were below the EORTC “All Myeloma Group” mean reference values for all symptoms. While MM participants reported above the mean EORTC-QLQ-C30 reference value for fatigue, SMM participants reported slightly less than the mean reference value. Contrastingly, for insomnia and appetite loss, SMM participants reported experiencing these symptoms at levels close to the mean EORTC-QLQ-C30 reference value (Fig. 4).

Across the entire sample, for both SMM and MM cohorts, the mean future perspective score was 52.4 and the mean disease symptoms score was 17.2. In the SMM group (*n* = 15), the mean future perspective score was 57.8 while the mean disease symptoms score was 20.7. This contrasted with the MM group in which the mean future perspective score was 38.9 while the mean disease symptoms score was 8.3. The higher mean future and disease scores in the SMM group reflected both higher symptomatology and better perspectives of the future in the SMM group as compared to the MM group.

### Cognitive interviewing of measures

A core part of the interview discussion was the completion and assessment of the EORTC QLQ-C30 and EORTC QLQ-MY20 instruments. Only SMM participants were asked cognitive interviewing questions to assess overall questionnaire comprehensiveness, instruction, response scale clarity, item comprehension, and to provide general feedback, as the instruments targeted SMM participants, in particular.

#### EORTC QLQ-C30 PRO questionnaire

After completing the EORTC QLQ-C30, SMM participants reported that instructions were easy to understand and agreed that a one-week recall period was appropriate. However, two SMM participants suggested that the recall period could be extended to a period of time that was ‘longer than a week’ because a one-month recall would account for symptom fluctuations.

Items were well understood by the study sample. Twenty percent of SMM participants (*n* = 3/15) mentioned that items within the EORTC QLQ-C30 could also apply to other conditions/comorbidities or external factors. But overall, SMM participants thought it was “comprehensive,” “pretty standard,” and “straightforward.” SMM participants felt it included questions that “painted a scientific and medical picture of the participant” and allowed them to think more about their condition.

#### EORTC QLQ-MY20 subscales

Overall, SMM participants expressed positive feedback about the ease of completion of the EORTC QLQ-MY20. Instructions were easy to understand, and participants felt the one-week time frame was an adequate recall period. SMM participants remarked that the questionnaire was “general,” “direct,” “straightforward,” consisted of “all fine questions,” and was “simple, very easy to answer.”

The only area of enhancement to the items recommended during the interviews was providing an open-text field to allow reporting on medications and other concerns the participant wishes to report. One participant pointed out that the items in the Future Perspective subscale could be a sensitive topic for some people with cancer (e.g., “Have you been worried about dying?” or “Have you worried about your health in the future?”).

Like SMM participant feedback on the EORTC QLQ-C30, three of the SMM participants mentioned difficulty in responding based on overall physical and mental capacities versus answering with SMM alone in mind. One participant requested “*if there would’ve been something in there stating if you responded to this question regarding SMM, what is your response versus if you’re responding to this question in response to your overall view regarding not taking into consideration just SMM. Again, I just think that might be more helpful in compiling your responses* (*58-year-old female*).”

## Discussion

This study is the first, to our knowledge, to report on the HRQoL of patients with SMM. Our study found meaningful symptom burden associated with this condition, even though it has largely been regarded as asymptomatic. Alongside the psychological burden of having a condition that may progress to MM, symptoms such as tiredness/fatigue, pain, and weakness were also frequently experienced. The more common symptoms reported by participants with SMM were the same symptoms experienced by MM patients. Overall, there was an average of 2.6 years since participants were diagnosed with SMM and tracking the progression of these symptoms over time could potentially help clinicians in assessing disease progression and whether treatment is needed. Report of the HRQoL impacts also exposed a greater picture of the SMM patient experience and provided insight into how the condition influenced their lives. Based on study findings, it was clear that SMM participants were particularly affected emotionally, physically, and in their daily lives.

Although this study was conducted with a small sample size, concept saturation was achieved. A perhaps more important limitation is that the sample was only English-speaking, and additional research is recommended to evaluate symptoms associated with SMM in non-English, non-US patient populations.

Additionally, PRO instruments are an important tool for increasing understanding of the SMM patient experience, and they should be incorporated in research where appropriate. The EORTC QLQ-C30, and nine items of the MY20 were found to be meaningful, relevant, and comprehensible to SMM patients, although at least one patient found the questions related to future perspective (regarding thoughts of death, for example) to be emotionally jarring. These instruments did not take long to administer and could be used in future SMM studies to assess HRQoL and symptoms specific to this condition. We did not find any evidence, in this sample, to suggest adding other symptoms to the list of those already being assessed by these instruments. The question as to whether these scales might be responsive to treatment effects in the context of a beneficial drug is worthy of further study. Such research could also elucidate meaningful change in PRO instrument scores for this SMM population. Further, although this study found the QLQ-C30 measure to be valid for administration among the SMM population, it must be noted that this is a general oncology PRO. This study found that this PRO alone may not account for comorbidities experienced by participants that are not oncology related. This becomes critical for consideration, especially in an older population.

The study population was carefully defined to ensure that participants were diagnosed with SMM but not MM. While it is possible that participants had undiagnosed MM at the time of the interview, participants were excluded if they had evidence of end-organ damage (e.g hypercalcemia, renal failure, anemia, and bone lesions) [9] or other MDE. In addition, none of the participants were receiving MM treatments at the time of the interview. The defined eligibility criteria were a strength of this research; however, it proved to be very difficult to recruit patients with SMM from clinical sites alone; therefore, we opted to use a recruitment vendor to recruit directly from physician practices. This could illustrate the current state of medical treatment for SMM, indicating that it is a medically underserved population.

## Conclusions

The interviews were a valid snapshot into the experiences of patients living with SMM. Most participants mentioned experiencing some level of tiredness/fatigue and weakness. Although generally considered an asymptomatic condition, this study indicates that many with SMM may experience one or more significant symptoms. The majority of SMM participants reported at least one way in which having SMM affected their lives, impacts that could be important to measure in a clinical trial. The top impact category most commonly reported was emotional impact, which was reported by 80% (*n* = 12/15) of participants. The items included in the EORTC QLQ-C30 and the nine items taken from the EORTC QLQ-MY20 provided good coverage of the symptom experience and the life impacts reported by patients with this condition. These findings suggest the need for increased surveillance of symptoms within the SMM population. and further suggest that the EORTC subscales can be used to assess symptoms and impacts in both the SMM and MM populations.

## Data Availability

The data that support the findings of this study are available from the corresponding author upon reasonable request.

## Abbreviations

EORTC: European Organisation for Research and Treatment of Cancer
HRQoL: Health-related quality of life
MDE: Myeloma-defining event
MGUC: Monoclonal gammopathy of undetermined significance
MM: Multiple myeloma
PRO: Patient-reported outcome
QLQ: Quality of Life Questionnaire
SMM: Smoldering multiple myeloma
